# Publisher Correction: Diversity of spatiotemporal coding reveals specialized visual processing streams in the mouse cortex

**DOI:** 10.1038/s41467-022-31464-4

**Published:** 2022-06-27

**Authors:** Xu Han, Ben Vermaercke, Vincent Bonin

**Affiliations:** 1 Neuro-Electronics Research Flanders, Kapeldreef 75, 3001 Leuven, Belgium; 2 KU Leuven, Department of Biology, 3000 Leuven, Belgium; 3 VIB-KU Leuven Center for Brain & Disease Research, 3000 Leuven, Belgium; 4 VIB, 3001 Leuven, Belgium; 5 imec, 3001 Leuven, Belgium; 6 KU Leuven, Leuven Brain Institute, 3000 Leuven, Belgium

**Keywords:** Striate cortex, Pattern vision, Sensory processing, Motion detection, Extrastriate cortex

Correction to: *Nature Communications* 10.1038/s41467-022-29656-z, published online 06 June 2022.

The original HTML version of this Article was updated shortly after publication because the previous HTML version linked to an incorrect Supplementary Movie file.

## Supplementary information

Updated Supplementary Movie

