# Supplementary material for: Diversity of spatiotemporal coding reveals specialized visual processing streams in the mouse cortex
Source: Nat Commun. 2022 Jun 6;13:3249. doi: 10.1038/s41467-022-29656-z (PMC9170684; doi:10.1038/s41467-022-29656-z)
Supplement: Supplementary file 1 — Supplementary Information [file 41467_2022_29656_MOESM1_ESM.pdf]

Diversity of spatiotemporal coding reveals specialized visual processing streams in the mouse cortex

SUPPLEMENTARY INFORMATION

Xu Han[1,2,*], Ben Vermaercke[1,3], Vincent Bonin[1,2,4,5,6,*]

[1] Neuro-Electronics Research Flanders, Kapeldreef 75, 3001 Leuven, Belgium.
[2] KU Leuven, Department of Biology & Leuven Brain Institute, 3000 Leuven, Belgium.
[3] VIB-KU Leuven Center for Brain & Disease Research, 3000 Leuven, Belgium.
[4] VIB, 3001 Leuven, Belgium.
[5] imec, 3001 Leuven, Belgium.
[6] Lead contact vincent.bonin@nerf.be
[*] For correspondence: xu.han@nerf.be (X.H.), vincent.bonin@nerf.be (V.B.).

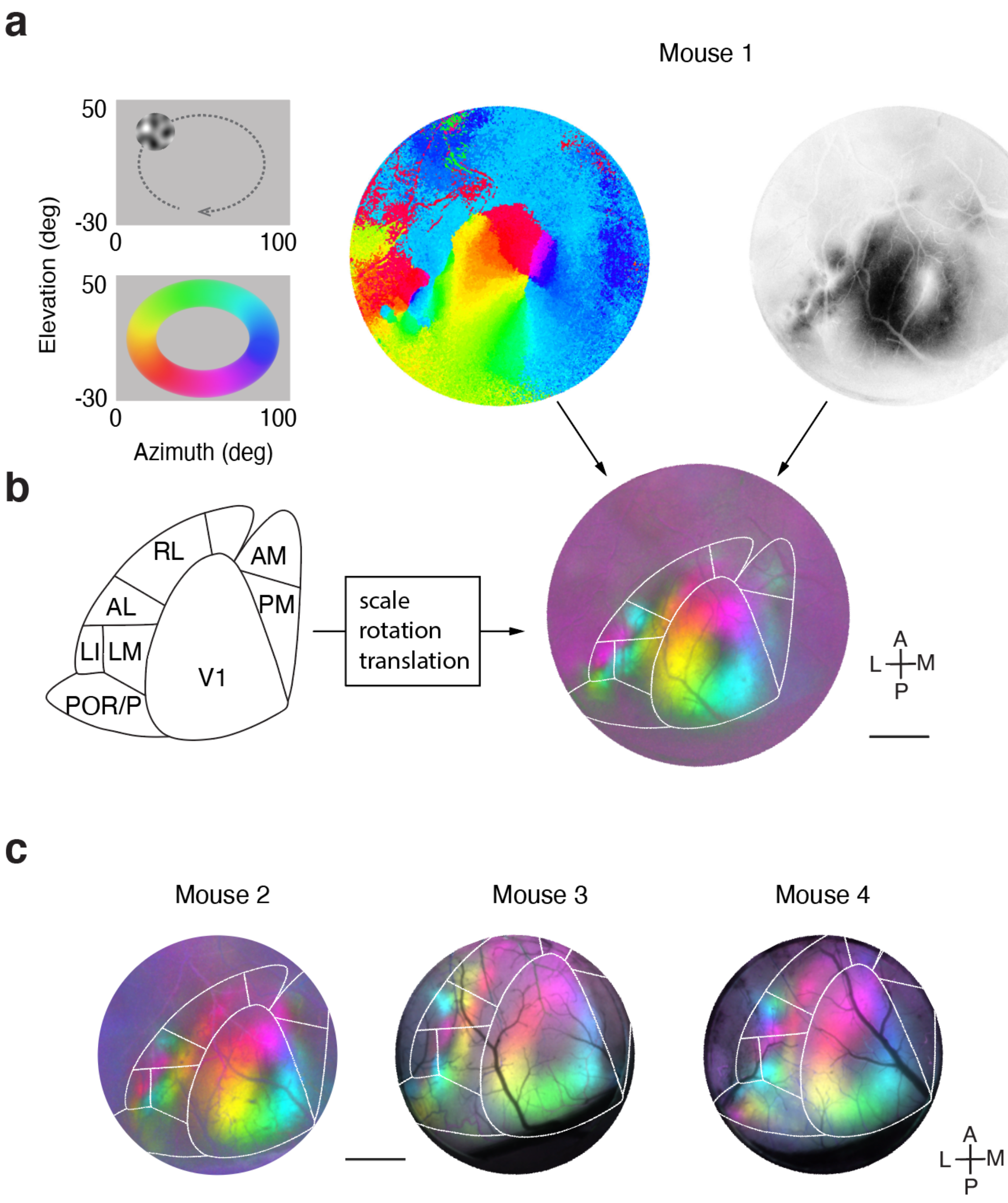

Supplementary Figure 1 Mapping of V1 and higher visual areas.

a Retinotopic mapping of an example mouse shown in Fig.1b. Left: The 'circling patch' visual stimulation paradigm and the covered visual spaces. Middle: a retinotopic map showing visual cortical regions (left panel) responding to different visual stimulation locations (left panel). Right: a response amplitude map showing the response strength of cortical regions to the circling patch stimuli. **b** A template visual cortical map was adapted and placed based on the retinotopy reversals and differences in response amplitudes between areas. **c** Other example retinotopic maps and area assignments. The cranial windows were 5 mm in diameter for mouse 1, and 4 mm for mice 2,3,4. Scale bar: 1 mm.

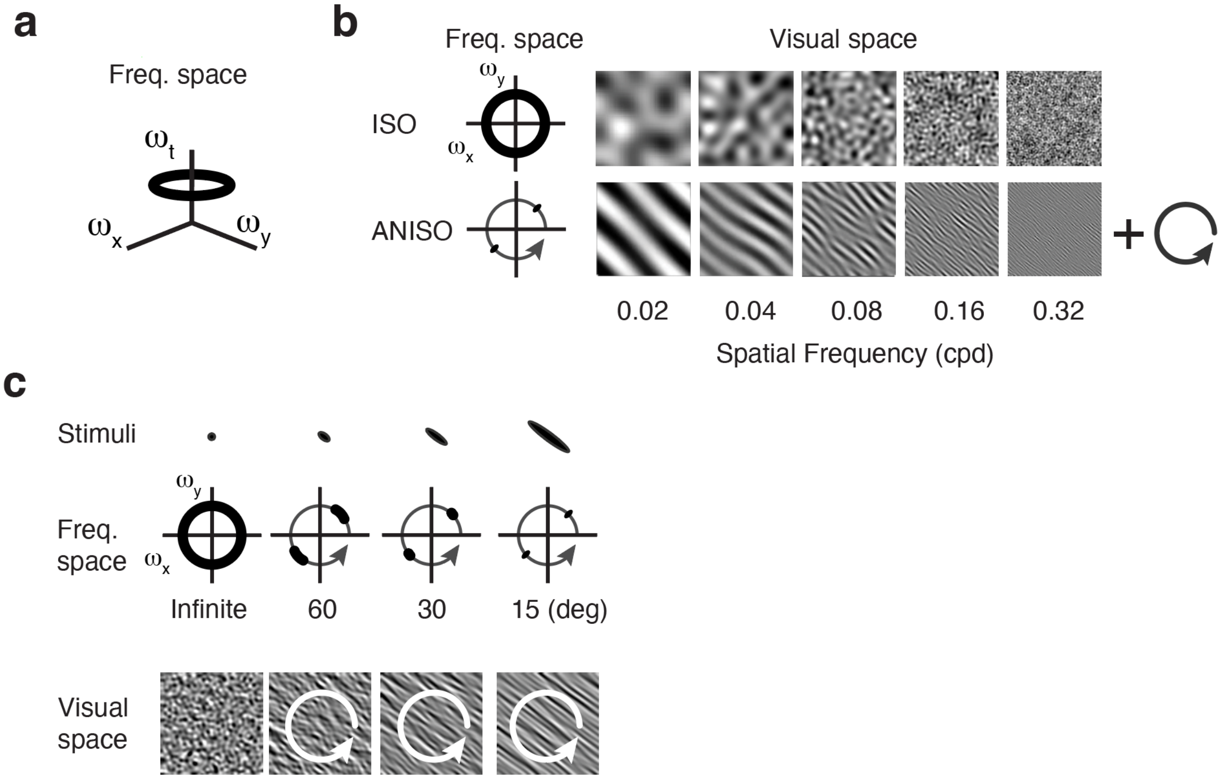

Supplementary Figure 2 Spatiotemporal noise stimuli.

a Scheme of the energy distribution of spatiotemporal bandpass noise stimuli in the 3D frequency space. Axis ω_x and ω_y define the 2D spatial frequency space. Axis ω_t defines the temporal frequency space. **b** Example images showing distinct spatial patterns of isotropic (ISO) and anisotropic (ANISO) stimuli. ISO stimuli were generated with 2D bandpass filters with various peak spatial frequencies (0.02, 0.04, 0.08, 0.16, 0.32 cpd; bandwidth 1 octave). ANISO stimuli have an additional angular filter (orientation bandwidth 15 deg) with the peak orientation varying from 0 to 180 deg during visual stimulation. **c** Example noise stimuli showing increasing stimulus anisotropies and texture elongation (bottom) corresponding to increasingly narrower orientation bandwidths (middle). Panel **c** is shown again in Fig. 6a.

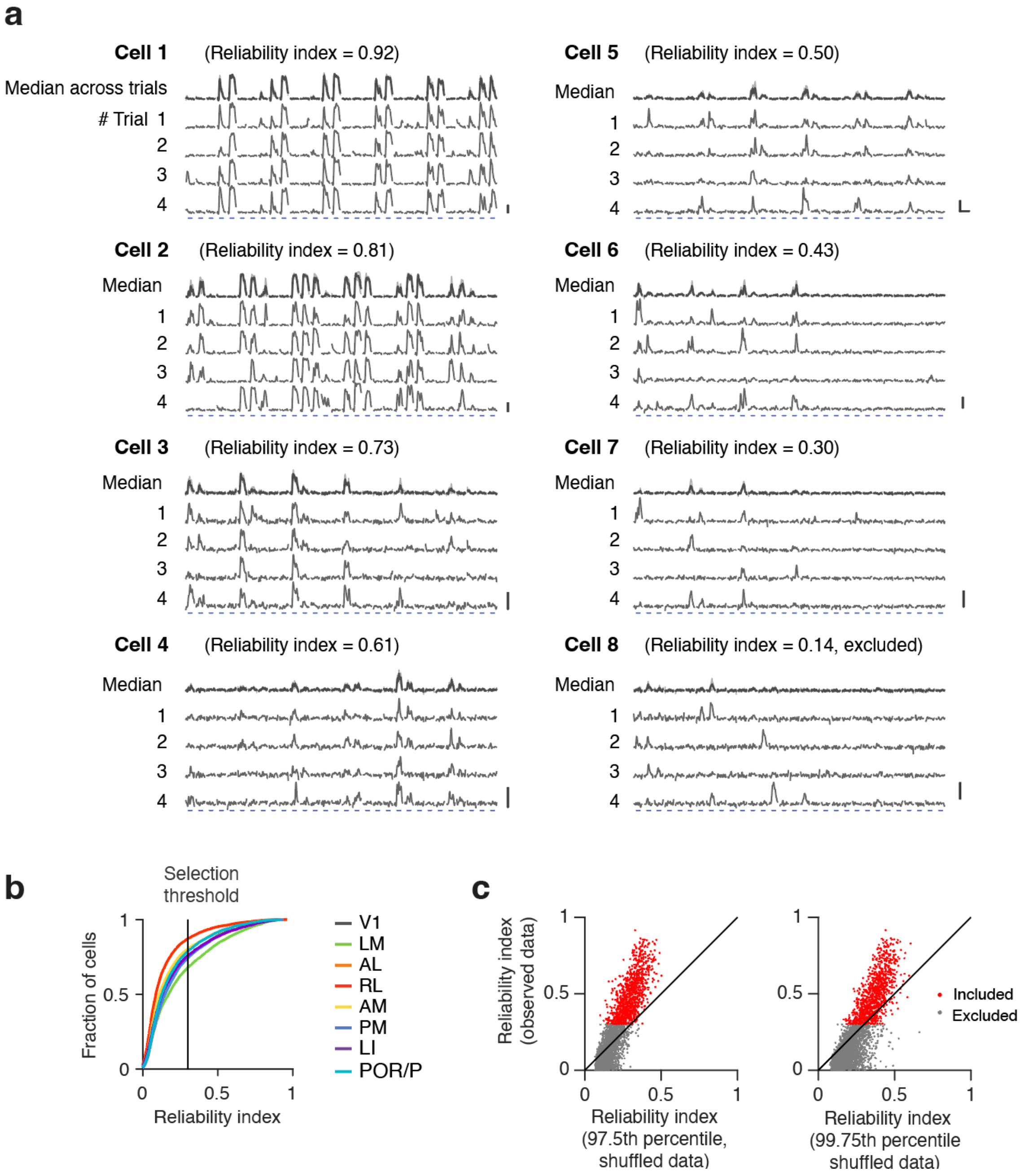

Supplementary Figure 3 Analysis of visual response reliability.

a Example cells showing responses across trials with different responsiveness and variabilities. The top trace of each cell shows the median responses across trials. The median absolute deviations across trials are shown as light gray shadows. Blue bars indicate 4s stimulus epochs. The reliability indices are measured as 75th percentile trial-to-trial correlation coefficients of response traces. Scale bars: 1 ΔF/F and 10 s. **b** Cumulative distributions of the response reliability index across the populations in different areas. **c** Comparison between cells' observed reliability indices (3000 random cells) with those computed from shuffled responses at 97.5 and 99.95 percentiles (1000 repetitions). Cells (red) with a reliability index above 0.3 were selected for further analysis.

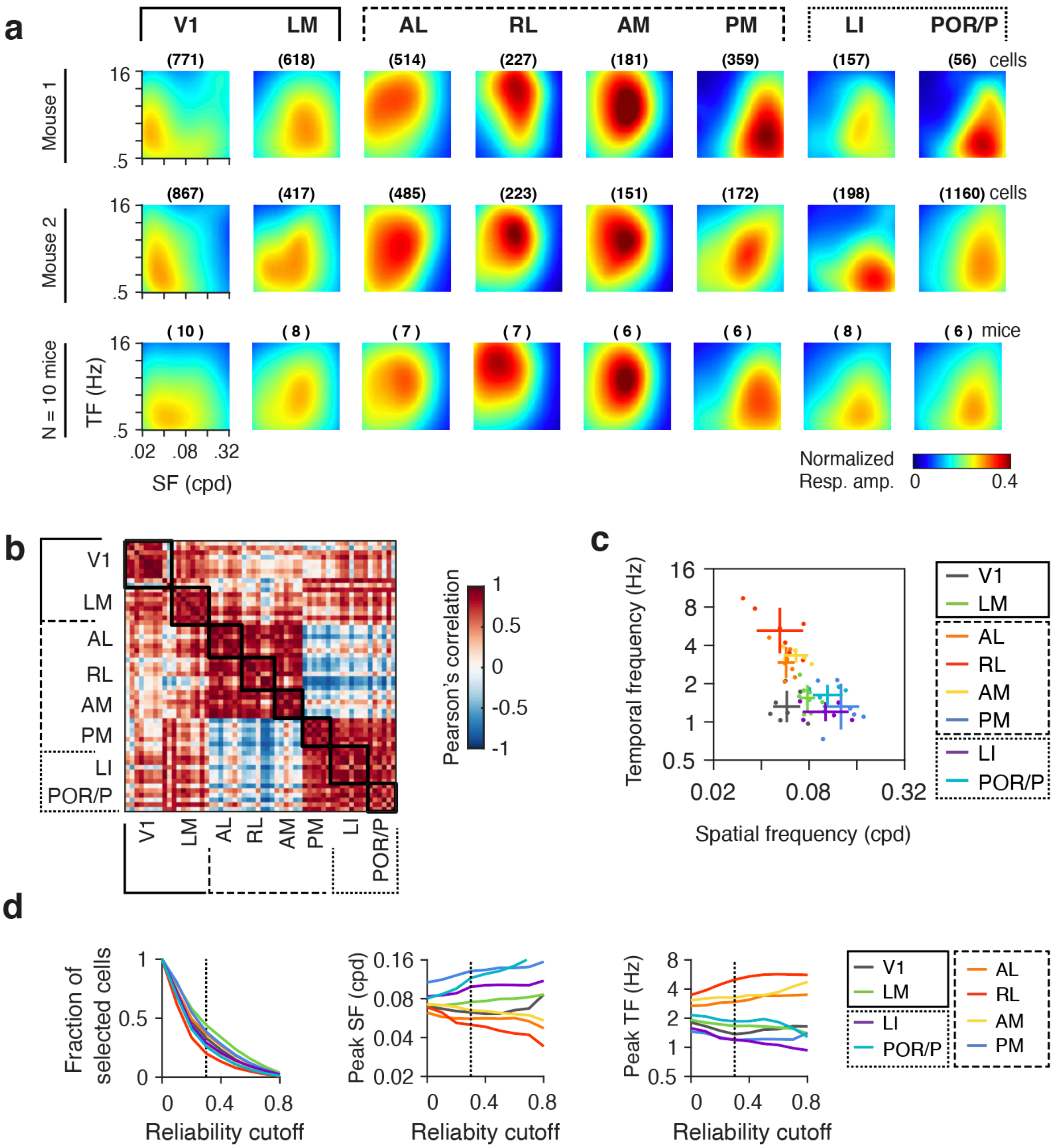

Supplementary Figure 4 Spatiotemporal representations are consistent and robust across mice.
a Population averages of spatiotemporal fits of different areas in example mouse 1 and 2, and the averaged surface across mice. Parentheses: # cells or mice. **b** Pearson's correlation coefficients between population averages of spatiotemporal fits of different areas and mice. The matrix is grouped and sorted by areas (black boxes). High coefficients within each box show high similarity across mice. **c** The distribution and variability of the mean peak spatiotemporal frequencies in different areas and animals. Dots represent the mean values of individual datasets. Bars: Mean ± s.e.m.. # of mice are shown in the parenthesis in **a**. **d** The impact of cell selection criteria on the tuning measurement. The left panel shows the fractions of neurons passing different thresholds of the response reliability index (*i.e.* reliability cutoff) in different areas. The middle and right panels show the median peak spatial and temporal frequencies with different cell selection thresholds in different areas. The vertical lines at 0.3 indicate the threshold used in this study.

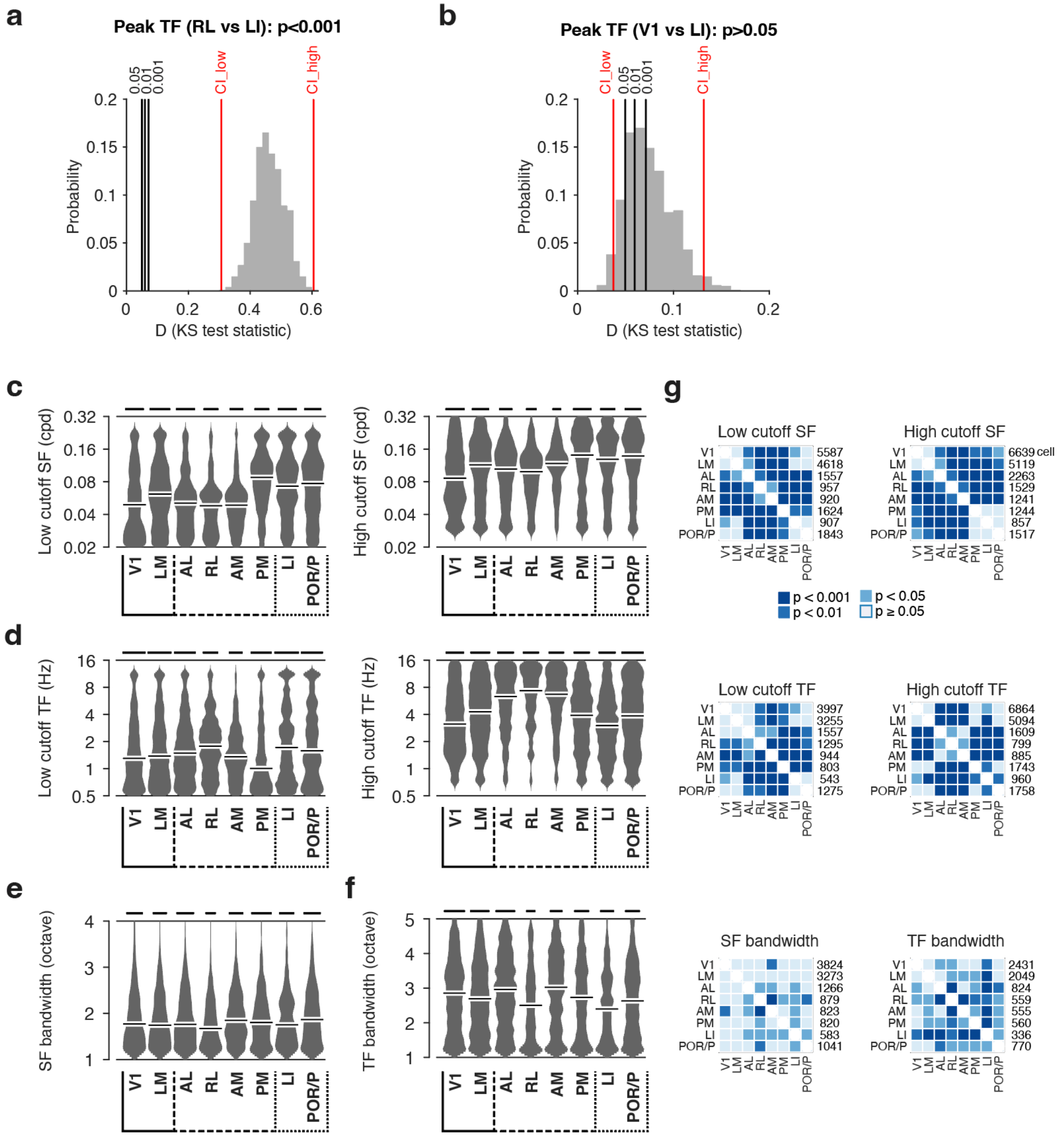

Supplementary Figure 5 Distributions of spatial and temporal bandwidth and cutoff frequencies.

a A demonstration of two-sided hierarchical bootstrap KS tests. The bootstrap KS tests were performed 1000 times. Each time, a resampled dataset was generated from each distribution using hierarchical bootstrapping (300 neurons per mouse, 5 mice per area, details in Methods). The two resampled datasets were compared using classic two-sample KS tests, which reported the differences in a *D* value. The distribution of bootstrap *D* values is shown in the histogram and is used to determine if the two distributions differ significantly. The histogram shows the distribution of bootstrap *D* values between area RL and LI in terms of the peak temporal frequencies (data shown in Fig. 3b left). The left and right thin red lines show the confidence intervals (a=0.001) of the bootstrap *D* values. The black vertical lines show the critical *D* values corresponding to a values (0.05, 0.01, and 0.001) respective. The lower bound of the confidence interval is larger than the critical *D* values corresponding to a=0.001, meaning the difference between the two populations is highly significant ($p < 0.001$). **b** Same as panel **a**, example statistical comparison showing no significant

difference ($p>0.05$) between area V1 and LI in terms of the distribution of peak temporal frequencies. The lower bound of confidence interval ($a=0.05$) is lower than the critical D values corresponding to $a=0.05$, suggesting no statistical differences between the two populations. **c** Distributions of low and high cutoff spatial frequencies across areas. For each distribution, Gaussian smooth kernel bandwidth: 0.2; bin size: 100; scale bar: 2.0% cells; black bars: median values. **d** Distributions of low and high cutoff spatial frequencies across areas. Same format as **c**. **e** Distributions of spatial frequency bandwidths across areas. Same format as **c**. **f** Distributions of temporal frequency bandwidths across areas. Same format as **e**. **g** Statistical differences between distributions. Two-sided hierarchical bootstrap KS tests.

SF: spatial frequency. TF: temporal frequency. BW: bandwidth.

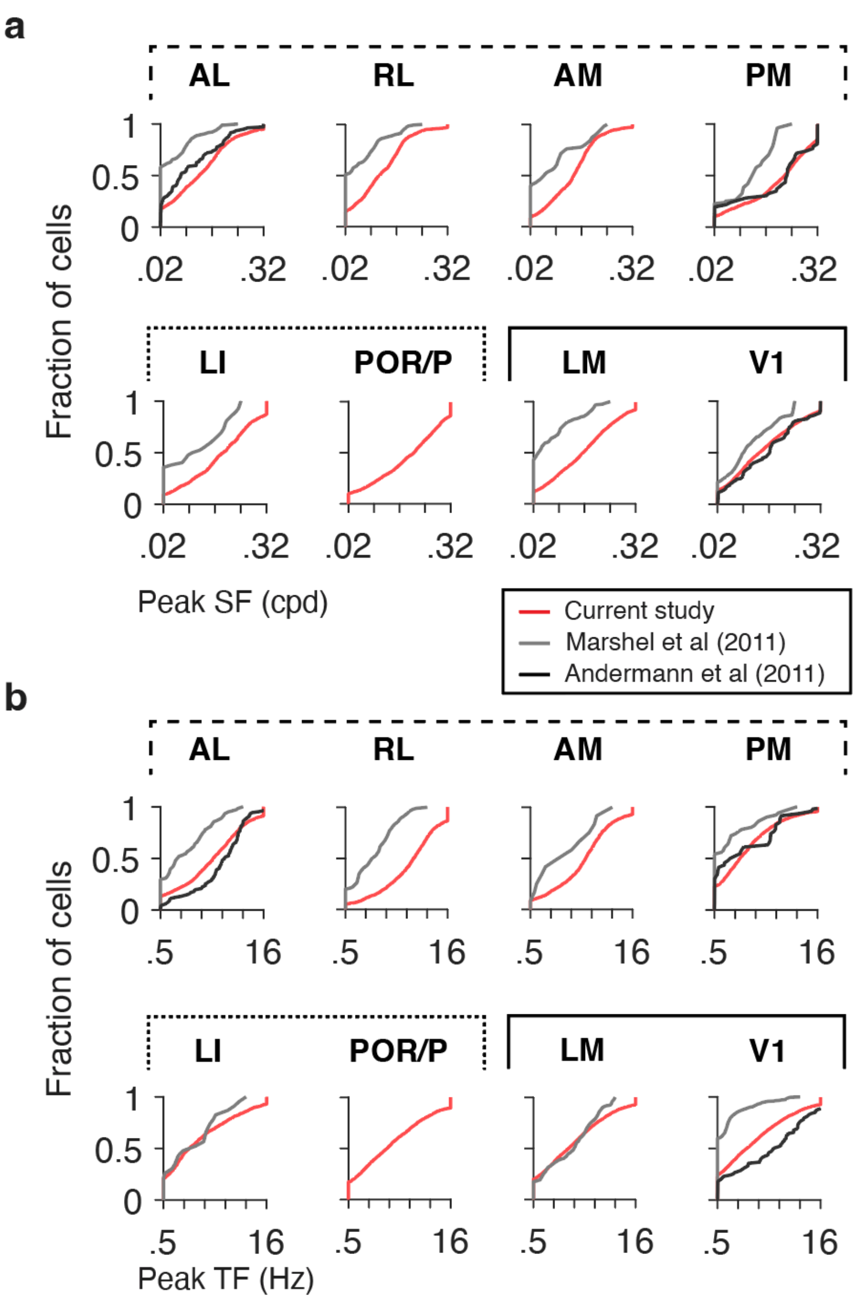

Supplementary Figure 6 Comparison of peak spatial and temporal frequencies across visual areas.

a Cumulative distributions of peak spatial frequencies reported in the current and two previous studies. The tuning measured in the current study is similar to those reported by Andermann et al., 2011, but show large shifts to higher frequencies in comparison to the work by Marshel et al., 2011. **b** Same style as **a**, for peak temporal frequency distributions.

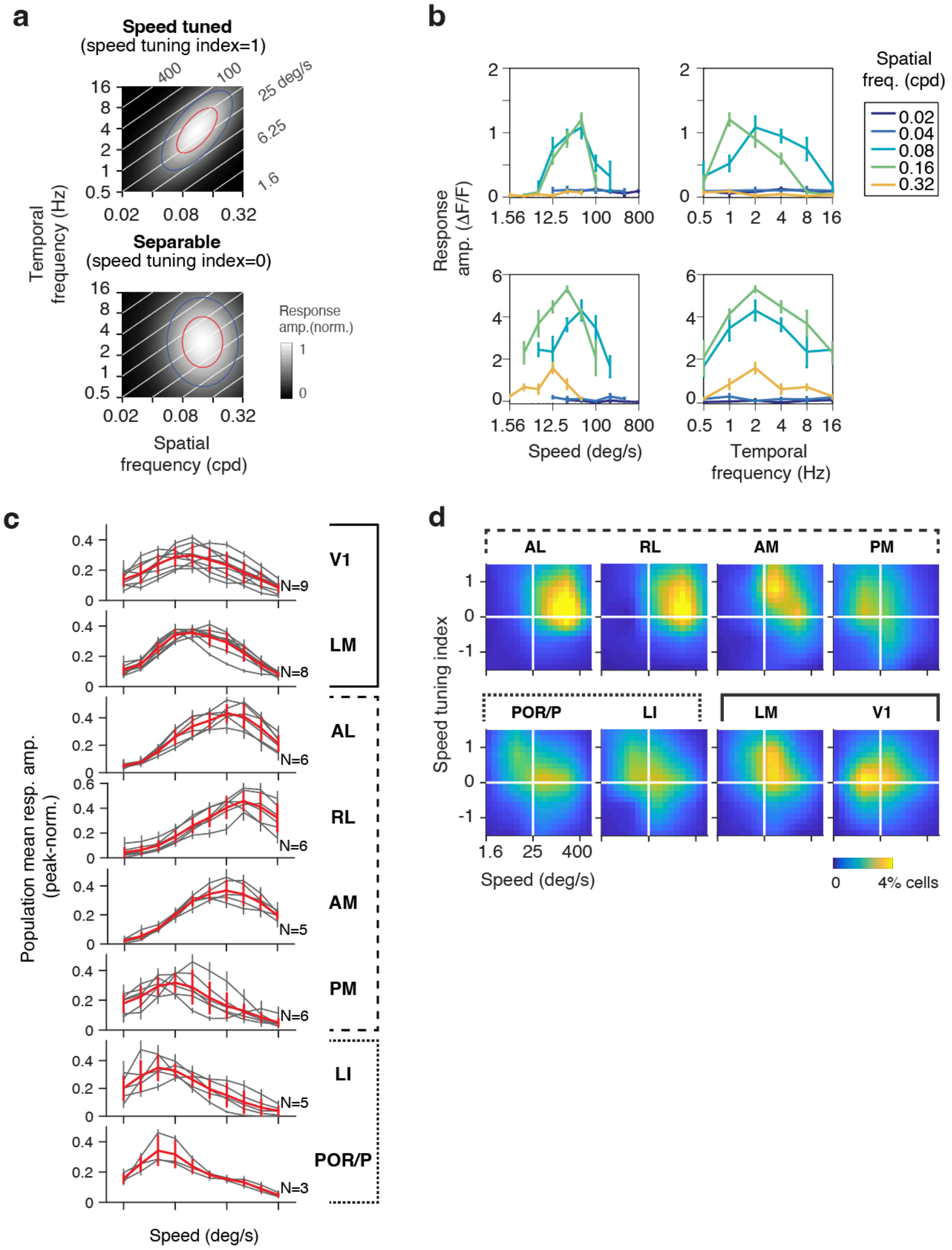

Supplementary Figure 7 Distinct speed tuning and preferences in dorsal and ventral areas.

a Example neurons that are speed tuned or untuned. The speed tuning index describes the interdependency between temporal frequency and spatial frequencies. If the index is close to 1, the cell is tuned for speed (upper left); if close to 0, the cell has separable tuning for spatial and temporal frequencies (red and blue contours show 80 and 50% peak amplitude; white lines indicate iso-speed lines). **b** Speed-tune cells have similar tuning curves for speed at different spatial frequencies (upper left), while the temporal frequency tuning curves change across spatial frequencies (upper right). Neurons with separable spatiotemporal tuning have similar tuning curves for temporal frequencies but not speed at different spatial frequencies (lower panels). Mean ± s.e.m.. n= 4 trials. **c** Average speed tuning curves in each area. Gray lines are the averages of peak-normalized responses to different speeds across the neuronal population in individual mice. Red lines are the averages across mice. Only showing mice with more than 20 speed tuned cells sampled. Mean ± s.e.m.. **d** Heatmaps of the density of cells in the space of peak speed and speed tuning index, showing no clear correlation between the peak speed and speed selectivity amongst neurons within each area.

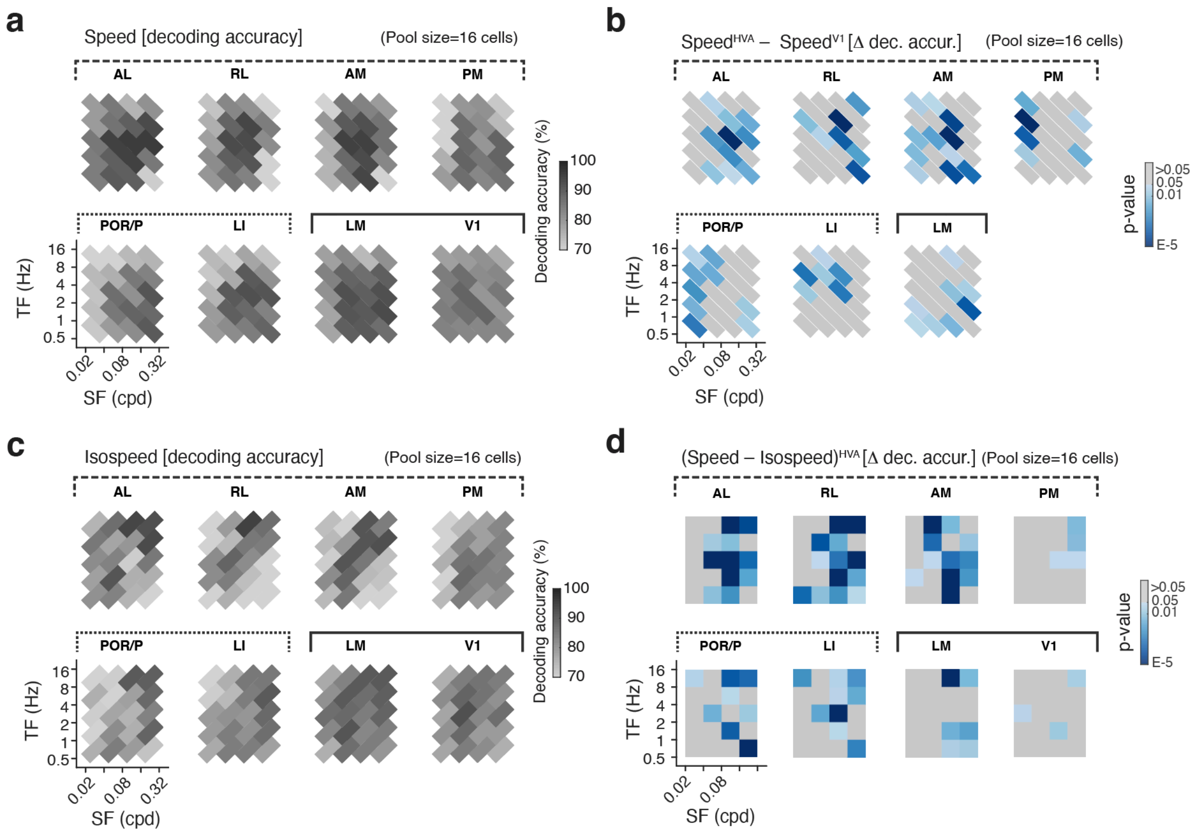

Supplementary Figure 8 Decoding analysis.

a Summary of the decoding accuracy for speed pairs in different areas with a pool size of 16 cells. Decoding accuracy is shown in line colors. 50% accuracy means the chance level. Panel V1 is also shown in **Fig. 4c** as a reference. **b** Summary of the statistical differences between higher visual areas and V1 in terms of the decoding accuracy of speed pairs, in relation to Fig. 4d. **c** Summary of the decoding accuracy for iso-speed pairs in different areas with a pool size of 16 cells. **d** Summary of the statistical differences between speed pairs and iso-speed pairs in terms of the decoding accuracy, in relation to Fig. 4f.

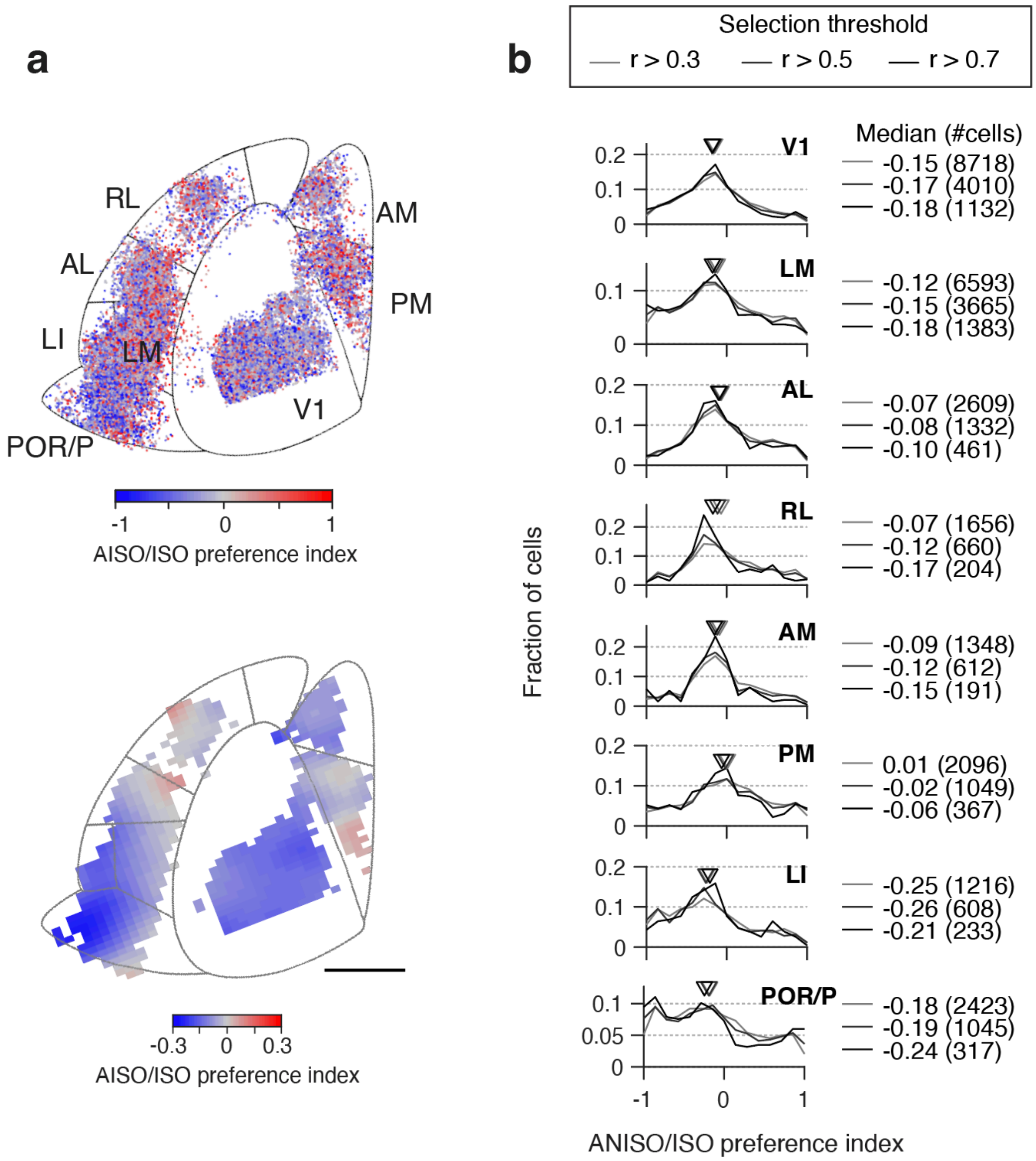

Supplementary Figure 9 ANISO/ISO preference is robust to changes in cell selection criterion.

a ANISO/ISO preference indices of characterized neurons (shown as dots in the left panel; dataset 1 and 2) on the cortical surface. Right: a spatially smoothed map showing the mean ANISO/ISO preference index across the cortical surface (Gaussian filter width 400 um). Scale bar: 1mm. **b** Distribution of ANISO/ISO preference indices with different cell selection thresholds (response reliability r >0.3, >0.5, or >0.7) in different areas. Median values are indicated with arrowheads.

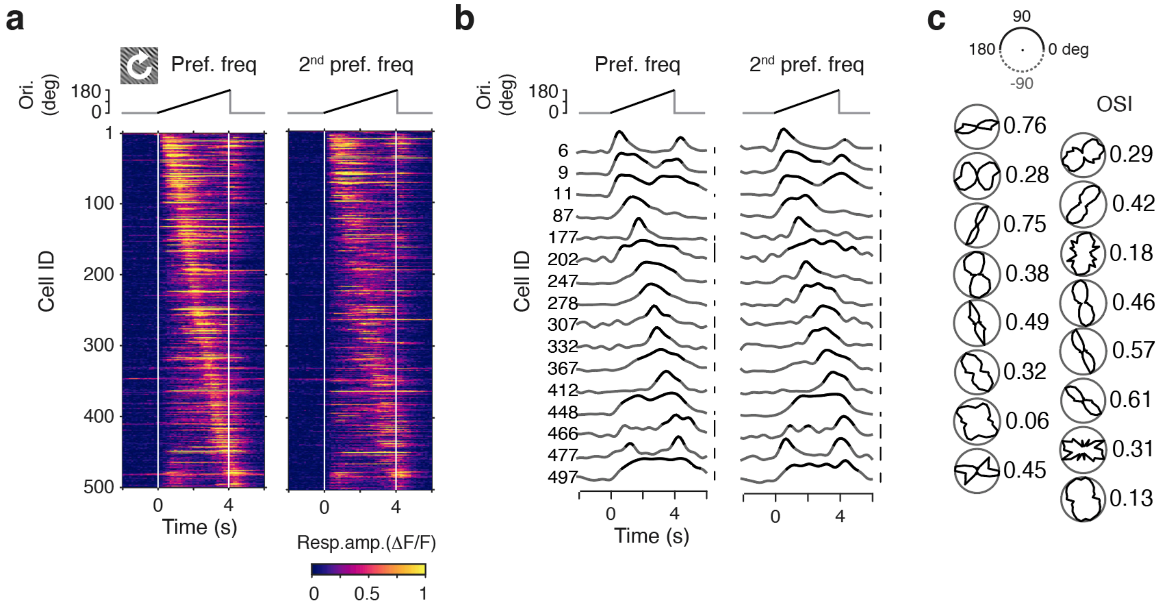

Supplementary Figure 10 Orientation responses are robust across stimuli of different scales.

a Population response matrices showing the calcium traces of 500 random V1 cells in response to ANISO stimuli with the preferred and second preferred frequencies (left and right columns), sorted according to the peak time of the highest responses. Vertical lines at 0 and 4 sec indicate the onset and offset of the visual stimulation. The top panels show the global orientation of rotary ANISO stimuli during the visual stimulation. These two matrices are highly correlated, suggesting these cells respond to specific orientations of the stimuli. **b** Example cells in **a**, showing comparable peak and 2nd peak responses. In each time course, the part above 50% of the peak amplitude is shown in black. Scale bar: 0.3 ΔF/F. **c** Polar plots showing orientation tuning properties of the example neurons in **b** (arranges from left to right, top to bottom). Black curves show peak-normalized calcium response amplitudes at different time bins of visual stimulation. The upper half of the polar plot is duplicated, rotated, and shown in the lower half, presented together to add visual ease. Orientation selectivity index (OSI), measured as 1-circular variance, is shown for each neuron.

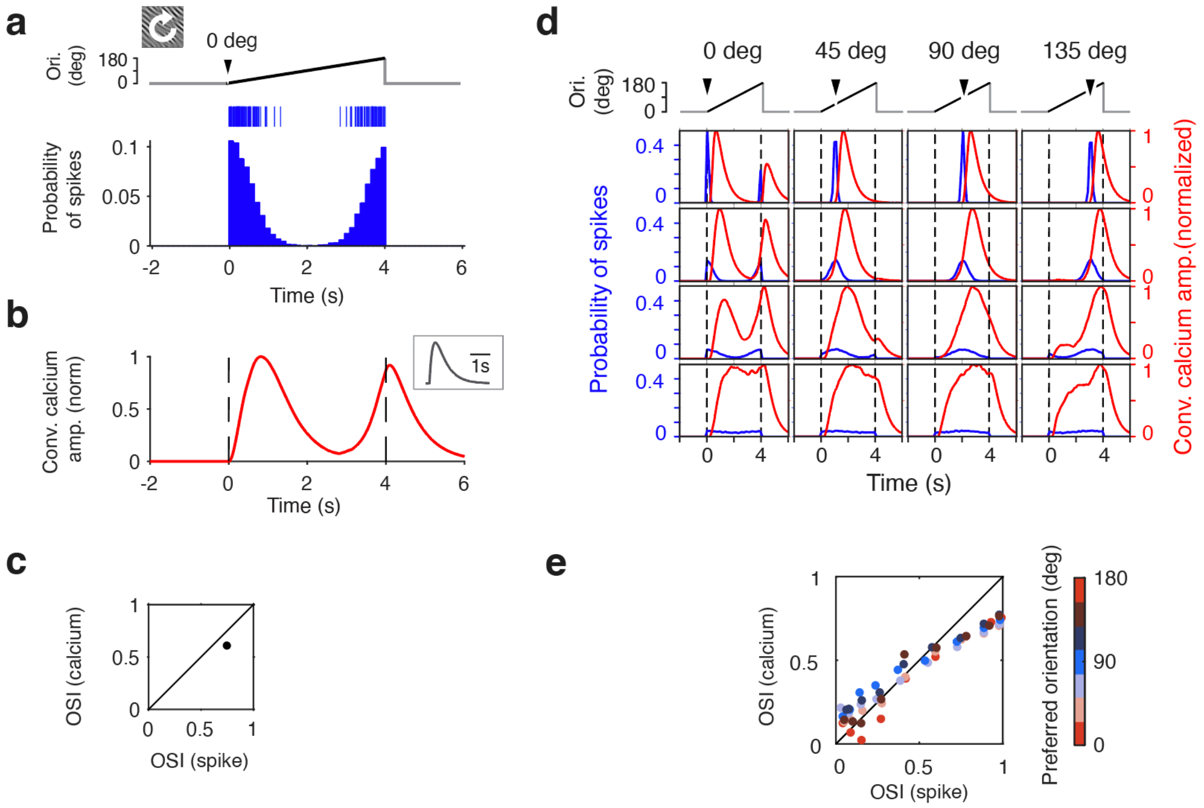

Supplementary Figure 11 Orientation selectivity index from simulated spikes and calcium traces.

a Histogram showing the probability of spikes of a model neuron in response to ANISO stimuli, with the corresponding random spike train shown in the above raster plot. The model cell prefers 0-degree orientation with a relatively sharp tuning (Gaussian distribution, FWHM 40 deg). 0 and 4 sec indicate the onset and offset of the visual stimulation. The top panels show the global orientation of rotary ANISO stimuli during the visual stimulation. Arrowhead: the preferred orientation of the mode neuron. **b** The resulting convoluted calcium trace by applying a kernel (inset) to the spike train shown in **a**. **c** Scatter plot showing the model cell's *OSI* computed from the spike train and from the calcium trace (see Methods). **d** Example model neurons with different preferred orientations (columns: 0, 45, 90, 135 deg) and tuning widths (rows: 10, 40, 90, 150 deg FWHM) in response to ANISO stimuli, showing the probability of spikes (blue curves) and convoluted calcium traces (red curves) as a function of orientation. **e** Scatter plot showing the *OSI* of neurons with various orientation tuning widths and preferred orientations, obtained from simulated spike trains and the corresponding convoluted calcium traces. Data points are color-coded by their preferred orientation. Comparing OSI^{spike} and OSI^{calc}, the two metrics showed a rough correspondence with an underestimation of OSI^{calc} for sharper tuning (tuning bandwidth < 70 deg FWHM).

FWHM: full-width-at-half-maximum. OSI: orientation selectivity index.

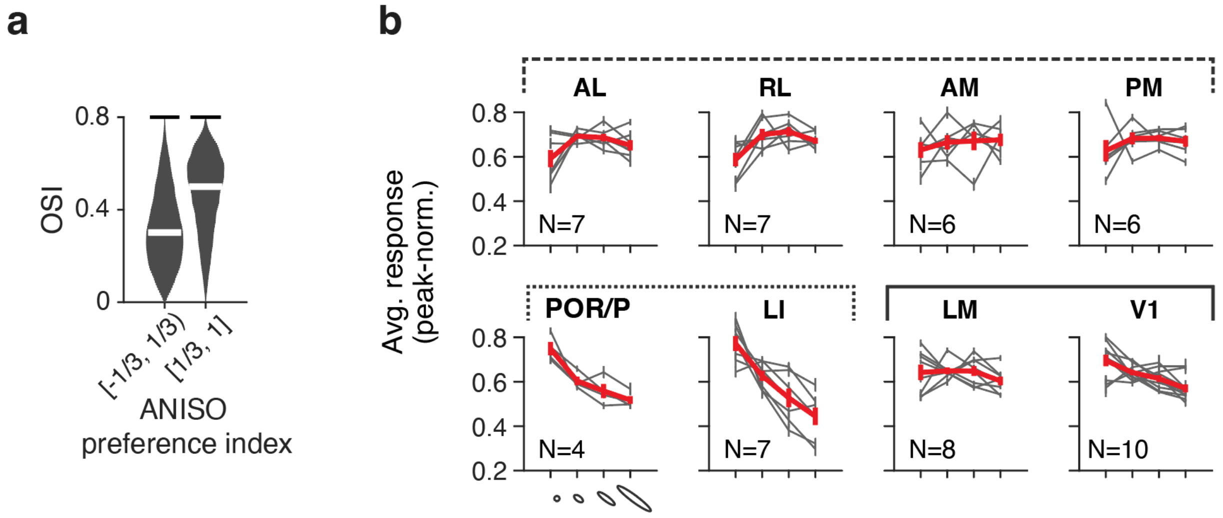

Supplementary Figure 12 Elongation tuning across mice.

a Distribution of OSI of neurons with intermediate and high API values (dataset3; 12143 vs. 7313 neurons). For each distribution: Gaussian smoothing kernel bandwidth 0.05; scale bar: 2% cells; white bars: median value. Two-sample KS tests, p<0.001. **b** Average tuning curves for stimulus elongation in different areas. Gary lines are the averages of peak-normalized responses to stimulus elongations across neurons in individual mice. Red lines are the averages across mice. Only showing mice with more than 50 reliable cells sampled. Mean ± s.e.m..

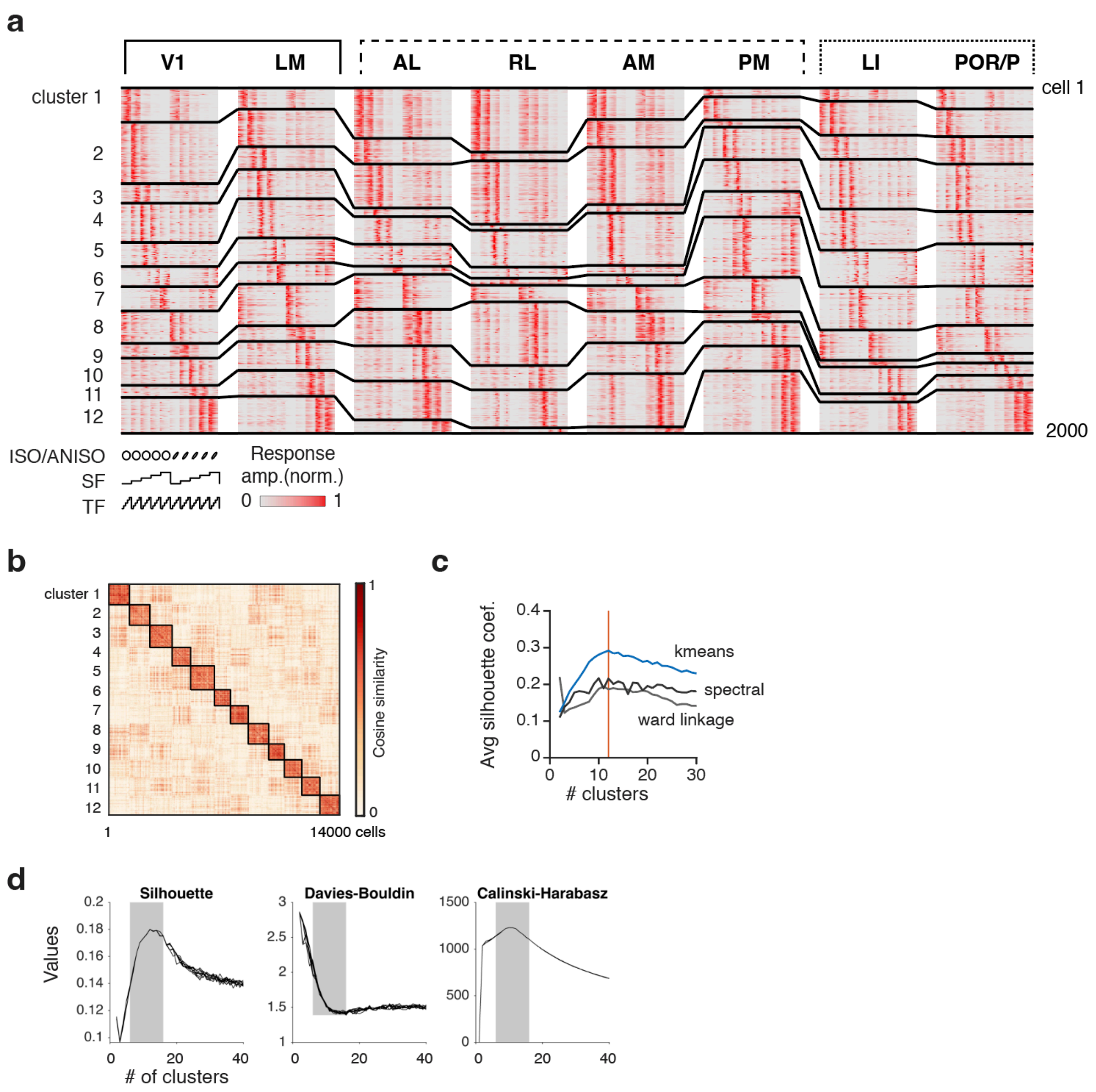

Supplementary Figure 13 Clustering analysis.

a Population response matrices showing that each visual cortical area is characterized by a distinct composition of functional cell types. Each matrix comprises the response profiles of 2000 randomly selected neurons. Each row represents a single cell's peak-normalized responses to ISO and ANISO stimuli. Cells were clustered into 12 functional groups based on the similarity of response profiles using a clustering approach. The black lines delineate neighboring groups and connect the same groups across matrices. Stimulus parameters are annotated below the V1 matrix. **b** Heatmap shows the cosine similarity matrix of the training dataset. Cells are grouped and sorted by clusters (in boxes). **c** Clustering analysis applied to datasets 1 and 2. Each curve indicates the mean silhouette coefficients as a function of the number of clusters using different clustering algorithms. The k-means clustering outperformed spectral clustering and ward linkage hierarchical clustering, suggesting an optimal separation of 12 clusters. **d** Additional metrics were used to narrow down the range of numbers of clusters in the k-means algorithm. The inflection points in different clustering performance evaluation metrics suggest similar ranges of proper numbers of clustering (gray areas). Different lines correspond to different initializations (n=10).

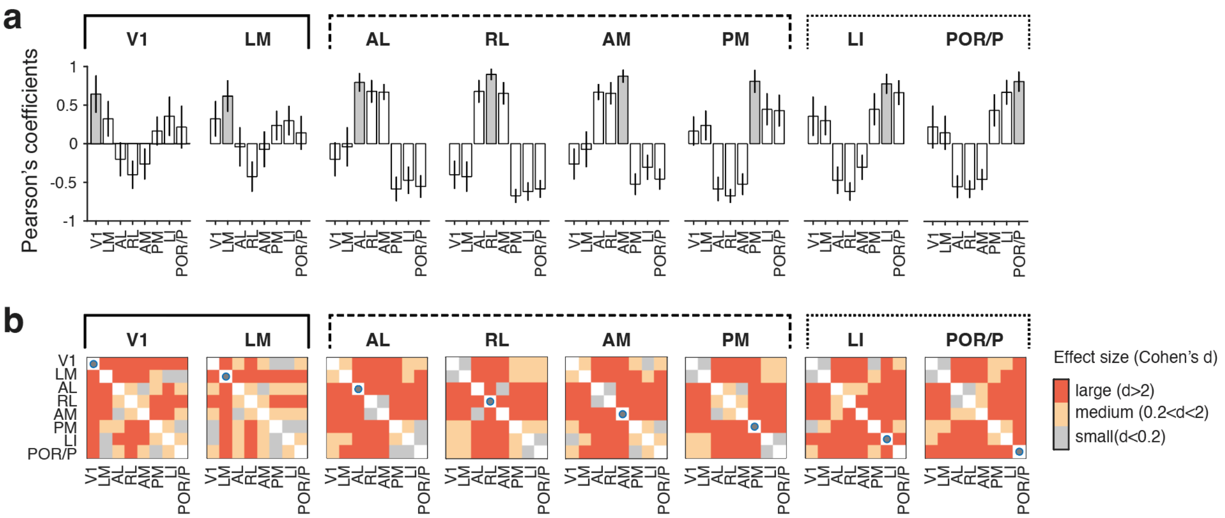

Supplementary Figure 14 Statistical analysis for the similarity between areas.

a Similarities between areas, measured as Pearson's correlation coefficients of individual areas' cellular compositions (fractions of functional clusters in Fig. 8e). A hierarchical bootstrapping approach (n=1000 iteration, 300 cells per mouse, 5 mice per area) was used to examine the variability across animals and subsamples. In each iteration, the similarity was calculated between different bootstrap subsamples of the same areas (gray bars) and between bootstrap subsamples between areas (white bars). Mean ± s.e.m.. **b** Statistical differences (i.e., effect size or *Cohen's d*) between the similarity estimates in the corresponding panels in **a**. One-sided bootstrap two-sample t-tests.
