# Supplementary material for: Diversity of spatiotemporal coding reveals specialized visual processing streams in the mouse cortex
Source: Nat Commun. 2022 Jun 6;13:3249. doi: 10.1038/s41467-022-29656-z (PMC9170684; doi:10.1038/s41467-022-29656-z)
Supplement: Supplementary file 3 — Description of Additional Supplementary Data Files [file 41467_2022_29656_MOESM3_ESM.pdf]

Descriptions of Additional Supplementary Data Files

Supplementary Movie 1: Functional mapping of visual cortical areas. Example functional mapping of visual cortical areas using circling patch stimuli in Thy1- GCaMP6 reporter mice. Widefield calcium imaging was performed through a cranial window in awake mice. As the stimulus patch circling on the display (left), corresponding portions of visual cortical areas were activated, showing calcium fluorescence increases (right). In each area, the activity spot moves along a circular trajectory, with the active spots in the neighboring areas rotating in the opposite direction. Area borders were drawn at where the moving spots converge and diverge. A sketch area map is aligned to retinotopic maps of individual mice.

Supplementary Movie 2: Visual noise stimuli. Example visual noise stimuli with different combinations of central spatial frequency, temporal frequency, and orientation bandwidths. The three stimulus sets, from left to right, were used to generate datasets 1-3, respectively.
